# (4*Z*)-4-[(2-Chloro­anilino)(phen­yl)methyl­idene]-3-methyl-1-phenyl-1*H*-pyrazol-5(4*H*)-one

**DOI:** 10.1107/S1600536812020004

**Published:** 2012-05-23

**Authors:** Li-Ying Xu, Ning Li, Jia-Min Li, Heng-Qiang Zhang, Zhen-Hai Sun

**Affiliations:** a School of Chemical Engineering and Technology, Harbin Institute of Technology, Harbin 150001, People’s Republic of China; b School of Science, Harbin University, Harbin 150080, People’s Republic of China; c School of Science, Northeast Forestry University, Harbin 150040, People’s Republic of China; d Department of Chemistry, East China Normal University, Shanghai 200062, People’s Republic of China

## Abstract

The title compound, C_23_H_18_ClN_3_O, exists in an enamine–keto form with the amino group involved in an intra­molecular N—H⋯O hydrogen bond. The five-membered ring is nearly planar, the largest deviation being 0.0004 (7) Å, and makes dihedral angles of 16.62 (6), 41.89 (5) and 71.27 (4)° with the phenyl rings. In the crystal, weak C—H⋯O hydrogen bonds link the mol­ecules into supra­molecular chains along the *b* axis.

## Related literature

For general background to Schiff bases derived from 1-phenyl-3-methyl-4-benzoyl-1*H*-pyrazol-5(4*H*)-one and their pharmaceutical and agrochemical applications, see: Casas *et al.* (2007 ▶); Zhang *et al.* (2008 ▶). For related structures, see: Zhang *et al.* (2007 ▶); Li *et al.* (2009 ▶); Chi *et al.* (2010 ▶).
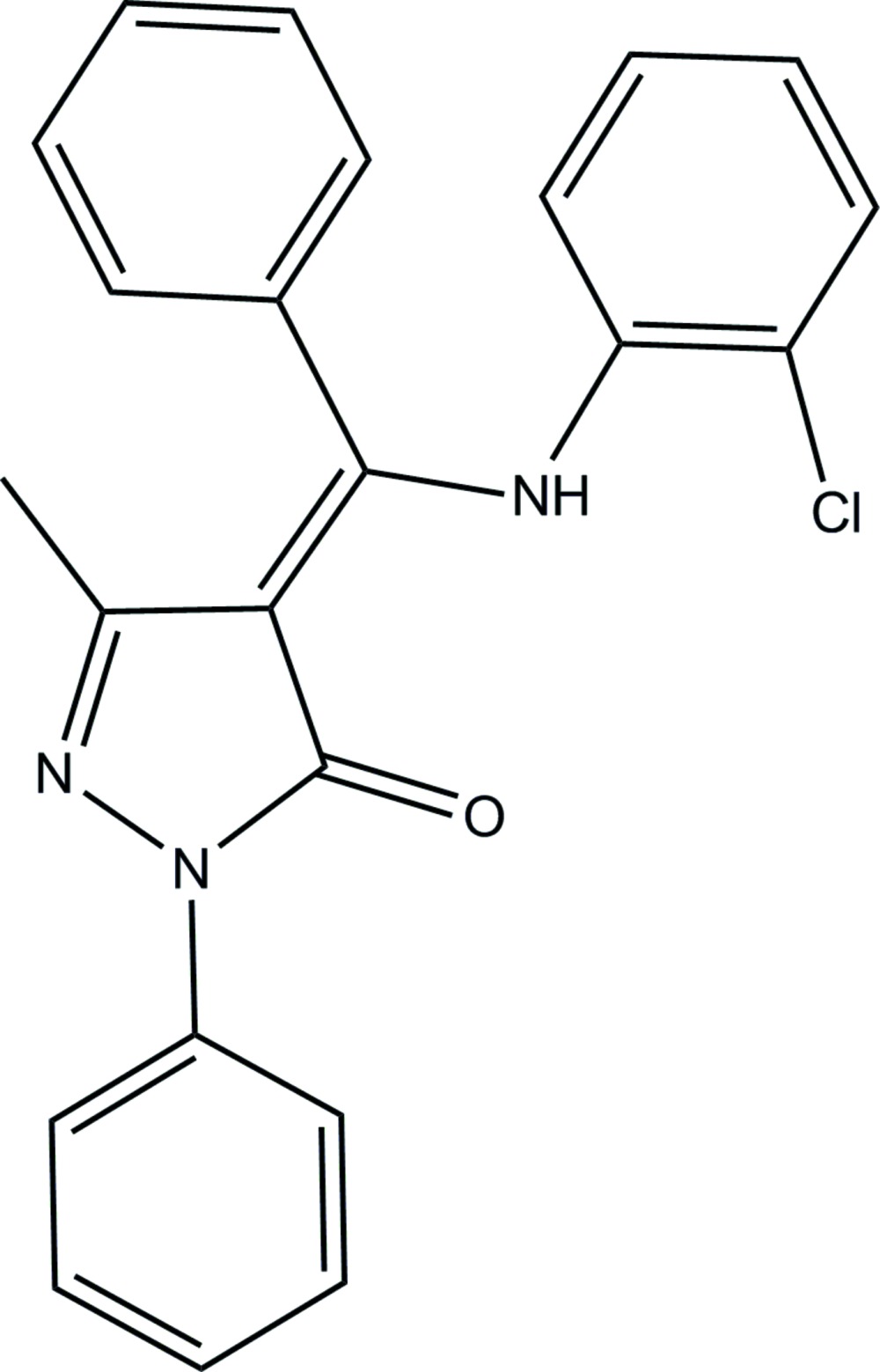


## Experimental

### 

#### Crystal data

C_23_H_18_ClN_3_O
*M* *_r_* = 387.85
Monoclinic, 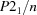

*a* = 9.0425 (3) Å
*b* = 18.5180 (7) Å
*c* = 11.1983 (4) Å
β = 90.423 (1)°
*V* = 1875.09 (12) Å^3^
*Z* = 4
Mo *K*α radiation
μ = 0.22 mm^−1^
*T* = 296 K
0.22 × 0.20 × 0.18 mm

#### Data collection

Bruker SMART CCD diffractometer
Absorption correction: multi-scan (*SADABS*; Sheldrick, 1996 ▶) *T*_min_ = 0.951, *T*_max_ = 0.959
17018 measured reflections
4637 independent reflections
4251 reflections with *I* > 2σ(*I*)
*R*_int_ = 0.013

#### Refinement

*R*[*F* ^2^ > 2σ(*F* ^2^)] = 0.034
*wR*(*F* ^2^) = 0.095
*S* = 1.04
4613 reflections
258 parameters
H atoms treated by a mixture of independent and constrained refinement
Δρ_max_ = 0.37 e Å^−3^
Δρ_min_ = −0.34 e Å^−3^

### 

Data collection: *SMART* (Bruker, 1998 ▶); cell refinement: *SAINT* (Bruker, 1998 ▶); data reduction: *SAINT*; program(s) used to solve structure: *SHELXS97* (Sheldrick, 2008 ▶); program(s) used to refine structure: *SHELXL97* (Sheldrick, 2008 ▶); molecular graphics: *SHELXTL* (Sheldrick, 2008 ▶); software used to prepare material for publication: *SHELXTL*.

## Supplementary Material

Crystal structure: contains datablock(s) I, global. DOI: 10.1107/S1600536812020004/zq2164sup1.cif

Structure factors: contains datablock(s) I. DOI: 10.1107/S1600536812020004/zq2164Isup2.hkl

Supplementary material file. DOI: 10.1107/S1600536812020004/zq2164Isup3.cml

Additional supplementary materials:  crystallographic information; 3D view; checkCIF report

## Figures and Tables

**Table 1:** Hydrogen-bond geometry (Å, °)

*D*—H⋯*A*	*D*—H	H⋯*A*	*D*⋯*A*	*D*—H⋯*A*
N3—H3*A*⋯O1	0.879 (18)	1.915 (18)	2.6800 (14)	144.5 (16)
C6—H6⋯O1	0.93	2.31	2.9027 (16)	121
C16—H16⋯O1^i^	0.93	2.56	3.4797 (17)	170

Symmetry code: (i) 
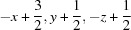
.

**Fig. 1.**
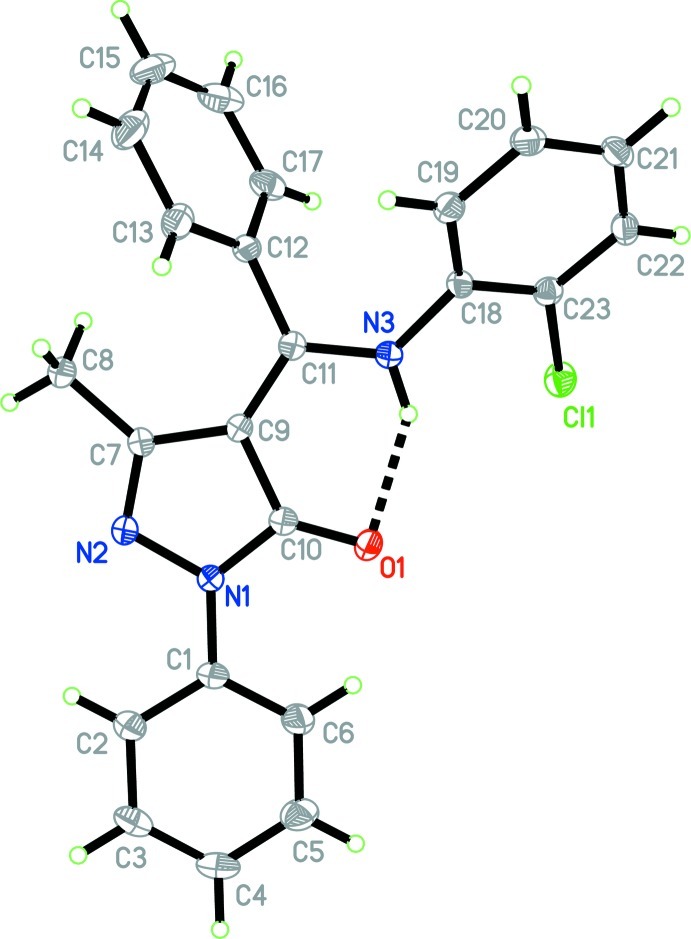
The molecular structure of the title compound (thermal ellipsoids are shown at the 30% probability level).

## supplementary crystallographic information

### Comment

The 1-phenyl-3-methyl-4-benzoyl-1*H*-pyrazol-5(4*H*)-ones (PMBP) are a novel type of β-enaminoketone. The Schiff bases derived from PMBP have attracted much attention due to their applications in pharmaceutical and agrochemical fields (*e.g.* Casas *et al.*, 2007; Zhang *et al.*, 2008). In order to expand this field, we now report the synthesis and structure of the title compound.

The molecular structure of the title compound is shown in Fig.1. Atoms O1, C10, C9 and C11 of the PMBP moiety and atom N3 of the *o*-chloroaniline group are coplanar, the largest deviation being 0.038 (11) Å for atom C10. The dihedral angle between this mean plane and the pyrazole ring of PMBP is 5.76 (3)°. The C9—C11 bond length of 1.3887 (15) Å, between usual C—C and C=C bond, indicates the delocalization of the electrons because of the addition of a proton to N3 is more favorable than to O2. The atom O2 of the PMBP moiety and the N3 atom of the *o*-chloroaniline group are on the same side of the C9—C11 bond, which are available for coordination with metal cations. A strong intramolecular hydrogen bond N3—H3A···O1 (Table 1) is also indicative of the enamine-keto form. In the crystal structure, the intramolecular hydrogen bond C6—H6···O1 and intermolecular hydrogen bond C16—H16···O1 are observed, the latter links the molecules into supramolecular chains along the *b* axis. All bond lengths and angles are normal and comparable with those found in related compounds (Zhang *et al.*, 2007; Li *et al.*, 2009; Chi *et al.*, 2010).

### Experimental

A mixture of a 10 ml PMBP (2 mmol, 0.5566 g) anhydrous ethanol solution, and a 0.21 ml of an *o*-chloroaniline (2 mmol, 0.2545 g) solution was refluxed for *ca* 5 h, with addition of a few drops of glacial acetic acid as a catalyst. The ethanol was removed by evaporation and the resulting green precipitate formed was filtered off, washed with cold anhydrous ethanol and dried in air. Yellow block single crystals suitable for analysis were obtained by slow evaporation of a solution in anhydrous ethanol at room temperature for a few days.

### Refinement

The H3A atom bonded to N3 was located in a difference Fourier map and refined freely. The other H atoms were placed in calculated positions, with C—H = 0.93 Å for phenyl, 0.96 Å for methyl H atoms, and refined as riding, with *U*_iso_(H) = 1.2*U*_eq_(C) for phenyl H, and 1.5*U*_eq_(C) for methyl H.

### Crystal data

**Table:**

C_23_H_18_ClN_3_O	*F*(000) = 808.0
*M**_r_* = 387.85	*D*_x_ = 1.374 Mg m^−^^3^
Monoclinic, *P*2_1_/*n*	Mo *K*α radiation, λ = 0.71073 Å
Hall symbol: -P2yn	Cell parameters from 9949 reflections
*a* = 9.0425 (3) Å	θ = 3.1–28.2°
*b* = 18.5180 (7) Å	µ = 0.22 mm^−^^1^
*c* = 11.1983 (4) Å	*T* = 296 K
β = 90.423 (1)°	Block, yellow
*V* = 1875.09 (12) Å^3^	0.22 × 0.20 × 0.18 mm
*Z* = 4	

### Data collection

**Table:**

Bruker SMART CCD diffractometer	4637 independent reflections
Radiation source: fine-focus sealed tube	4251 reflections with *I* > 2σ(*I*)
Graphite monochromator	*R*_int_ = 0.013
ω scans	θ_max_ = 28.2°, θ_min_ = 2.1°
Absorption correction: multi-scan (*SADABS*; Sheldrick, 1996)	*h* = −11→12
*T*_min_ = 0.951, *T*_max_ = 0.959	*k* = −24→23
17018 measured reflections	*l* = −14→14

### Refinement

**Table:**

Refinement on *F*^2^	Primary atom site location: structure-invariant direct methods
Least-squares matrix: full	Secondary atom site location: difference Fourier map
*R*[*F*^2^ > 2σ(*F*^2^)] = 0.034	Hydrogen site location: inferred from neighbouring sites
*wR*(*F*^2^) = 0.095	H atoms treated by a mixture of independent and constrained refinement
*S* = 1.04	*w* = 1/[σ^2^(*F*_o_^2^) + (0.0482*P*)^2^ + 0.9306*P*] where *P* = (*F*_o_^2^ + 2*F*_c_^2^)/3
4613 reflections	(Δ/σ)_max_ < 0.001
258 parameters	Δρ_max_ = 0.37 e Å^−^^3^
0 restraints	Δρ_min_ = −0.34 e Å^−^^3^

### Special details

**Table:**

Geometry. All e.s.d.'s (except the e.s.d. in the dihedral angle between two l.s. planes) are estimated using the full covariance matrix. The cell e.s.d.'s are taken into account individually in the estimation of e.s.d.'s in distances, angles and torsion angles; correlations between e.s.d.'s in cell parameters are only used when they are defined by crystal symmetry. An approximate (isotropic) treatment of cell e.s.d.'s is used for estimating e.s.d.'s involving l.s. planes.
Refinement. Refinement of *F*^2^ against ALL reflections. The weighted *R*-factor *wR* and goodness of fit *S* are based on *F*^2^, conventional *R*-factors *R* are based on *F*, with *F* set to zero for negative *F*^2^. The threshold expression of *F*^2^ > σ(*F*^2^) is used only for calculating *R*-factors(gt) *etc*. and is not relevant to the choice of reflections for refinement. *R*-factors based on *F*^2^ are statistically about twice as large as those based on *F*, and *R*- factors based on ALL data will be even larger.

### Fractional atomic coordinates and isotropic or equivalent isotropic displacement parameters (Å^2^)

**Table:**

	*x*	*y*	*z*	*U*_iso_*/*U*_eq_	
C1	0.86725 (13)	−0.06692 (6)	−0.09829 (10)	0.0186 (2)	
C2	0.87873 (15)	−0.07367 (7)	−0.22145 (11)	0.0238 (2)	
H2	0.8476	−0.0364	−0.2713	0.029*	
C3	0.93725 (15)	−0.13668 (7)	−0.26969 (12)	0.0271 (3)	
H3	0.9454	−0.1413	−0.3521	0.033*	
C4	0.98343 (14)	−0.19250 (7)	−0.19629 (12)	0.0253 (3)	
H4	1.0181	−0.2354	−0.2291	0.030*	
C5	0.97757 (15)	−0.18398 (7)	−0.07385 (12)	0.0284 (3)	
H5	1.0116	−0.2208	−0.0242	0.034*	
C6	0.92153 (15)	−0.12112 (7)	−0.02397 (11)	0.0260 (3)	
H6	0.9203	−0.1153	0.0585	0.031*	
C7	0.70636 (14)	0.10296 (6)	−0.05496 (10)	0.0196 (2)	
C8	0.67068 (17)	0.17489 (7)	−0.10804 (11)	0.0269 (3)	
H8A	0.7155	0.1787	−0.1852	0.040*	
H8B	0.7083	0.2124	−0.0570	0.040*	
H8C	0.5654	0.1798	−0.1161	0.040*	
C9	0.68060 (13)	0.07593 (6)	0.06368 (10)	0.0187 (2)	
C10	0.74394 (13)	0.00384 (6)	0.06478 (10)	0.0198 (2)	
C11	0.62430 (12)	0.10790 (6)	0.16590 (10)	0.0164 (2)	
C12	0.56486 (12)	0.18268 (6)	0.16522 (10)	0.0169 (2)	
C13	0.43881 (14)	0.20000 (7)	0.09874 (11)	0.0234 (2)	
H13	0.3877	0.1641	0.0577	0.028*	
C14	0.38969 (16)	0.27111 (8)	0.09393 (13)	0.0326 (3)	
H14	0.3046	0.2826	0.0509	0.039*	
C15	0.46709 (18)	0.32468 (7)	0.15296 (14)	0.0359 (3)	
H15	0.4353	0.3723	0.1478	0.043*	
C16	0.59174 (17)	0.30782 (7)	0.21975 (13)	0.0318 (3)	
H16	0.6434	0.3442	0.2594	0.038*	
C17	0.63988 (14)	0.23657 (7)	0.22775 (11)	0.0225 (2)	
H17	0.7218	0.2250	0.2747	0.027*	
C18	0.57642 (12)	0.08798 (6)	0.38216 (9)	0.0161 (2)	
C19	0.43971 (13)	0.12143 (6)	0.39789 (10)	0.0189 (2)	
H19	0.3851	0.1365	0.3317	0.023*	
C20	0.38483 (13)	0.13232 (6)	0.51203 (11)	0.0211 (2)	
H20	0.2938	0.1548	0.5218	0.025*	
C21	0.46478 (14)	0.10990 (6)	0.61183 (10)	0.0213 (2)	
H21	0.4270	0.1172	0.6879	0.026*	
C22	0.60091 (13)	0.07666 (6)	0.59773 (10)	0.0196 (2)	
H22	0.6550	0.0616	0.6642	0.024*	
C23	0.65570 (12)	0.06605 (6)	0.48371 (10)	0.0166 (2)	
Cl1	0.82565 (3)	0.023579 (16)	0.46642 (3)	0.02280 (9)	
H3A	0.678 (2)	0.0288 (10)	0.2610 (16)	0.034 (5)*	
N1	0.79928 (12)	−0.00523 (5)	−0.04860 (8)	0.0200 (2)	
N2	0.77460 (12)	0.05536 (5)	−0.12101 (9)	0.0210 (2)	
N3	0.63332 (12)	0.07069 (6)	0.26879 (8)	0.0194 (2)	
O1	0.74879 (11)	−0.04063 (5)	0.14826 (8)	0.0267 (2)	

### Atomic displacement parameters (Å^2^)

**Table:**

	*U*^11^	*U*^22^	*U*^33^	*U*^12^	*U*^13^	*U*^23^
C1	0.0193 (5)	0.0172 (5)	0.0194 (5)	0.0015 (4)	0.0010 (4)	−0.0038 (4)
C2	0.0306 (6)	0.0216 (6)	0.0193 (5)	0.0019 (5)	0.0034 (5)	−0.0022 (4)
C3	0.0305 (6)	0.0278 (7)	0.0232 (6)	0.0001 (5)	0.0044 (5)	−0.0099 (5)
C4	0.0191 (5)	0.0218 (6)	0.0349 (7)	0.0013 (4)	0.0014 (5)	−0.0117 (5)
C5	0.0285 (6)	0.0243 (6)	0.0322 (7)	0.0094 (5)	−0.0021 (5)	−0.0023 (5)
C6	0.0308 (6)	0.0258 (6)	0.0214 (6)	0.0095 (5)	−0.0013 (5)	−0.0023 (5)
C7	0.0268 (6)	0.0176 (5)	0.0143 (5)	0.0023 (4)	0.0000 (4)	0.0007 (4)
C8	0.0436 (7)	0.0197 (6)	0.0177 (5)	0.0079 (5)	0.0046 (5)	0.0039 (4)
C9	0.0255 (6)	0.0154 (5)	0.0152 (5)	0.0036 (4)	0.0005 (4)	0.0012 (4)
C10	0.0256 (6)	0.0175 (5)	0.0164 (5)	0.0044 (4)	0.0019 (4)	0.0001 (4)
C11	0.0179 (5)	0.0160 (5)	0.0152 (5)	0.0010 (4)	−0.0007 (4)	0.0006 (4)
C12	0.0195 (5)	0.0155 (5)	0.0158 (5)	0.0015 (4)	0.0042 (4)	0.0011 (4)
C13	0.0227 (6)	0.0242 (6)	0.0232 (6)	0.0036 (5)	0.0015 (4)	0.0052 (4)
C14	0.0303 (7)	0.0321 (7)	0.0356 (7)	0.0139 (6)	0.0092 (5)	0.0139 (6)
C15	0.0444 (8)	0.0186 (6)	0.0452 (8)	0.0103 (6)	0.0247 (7)	0.0082 (6)
C16	0.0402 (8)	0.0192 (6)	0.0362 (7)	−0.0072 (5)	0.0213 (6)	−0.0073 (5)
C17	0.0229 (6)	0.0225 (6)	0.0222 (5)	−0.0036 (4)	0.0078 (4)	−0.0049 (4)
C18	0.0201 (5)	0.0140 (5)	0.0143 (5)	−0.0007 (4)	0.0023 (4)	0.0001 (4)
C19	0.0202 (5)	0.0171 (5)	0.0193 (5)	0.0009 (4)	0.0017 (4)	0.0016 (4)
C20	0.0222 (5)	0.0159 (5)	0.0251 (6)	0.0001 (4)	0.0074 (4)	−0.0003 (4)
C21	0.0290 (6)	0.0176 (5)	0.0175 (5)	−0.0040 (4)	0.0077 (4)	−0.0023 (4)
C22	0.0263 (6)	0.0174 (5)	0.0151 (5)	−0.0040 (4)	−0.0003 (4)	0.0007 (4)
C23	0.0167 (5)	0.0149 (5)	0.0182 (5)	−0.0015 (4)	0.0008 (4)	0.0005 (4)
Cl1	0.01928 (14)	0.02670 (16)	0.02240 (15)	0.00267 (10)	−0.00047 (10)	0.00295 (10)
N1	0.0294 (5)	0.0163 (5)	0.0142 (4)	0.0054 (4)	0.0020 (4)	0.0006 (3)
N2	0.0311 (5)	0.0170 (5)	0.0150 (4)	0.0037 (4)	0.0003 (4)	0.0018 (4)
N3	0.0254 (5)	0.0179 (5)	0.0148 (4)	0.0072 (4)	0.0028 (4)	0.0007 (3)
O1	0.0408 (5)	0.0208 (4)	0.0186 (4)	0.0113 (4)	0.0073 (4)	0.0054 (3)

### Geometric parameters (Å, º)

**Table:**

C1—C2	1.3895 (16)	C12—C13	1.3941 (16)
C1—C6	1.3910 (17)	C13—C14	1.3906 (18)
C1—N1	1.4133 (14)	C13—H13	0.9300
C2—C3	1.3920 (17)	C14—C15	1.380 (2)
C2—H2	0.9300	C14—H14	0.9300
C3—C4	1.383 (2)	C15—C16	1.384 (2)
C3—H3	0.9300	C15—H15	0.9300
C4—C5	1.3816 (19)	C16—C17	1.3921 (19)
C4—H4	0.9300	C16—H16	0.9300
C5—C6	1.3885 (18)	C17—H17	0.9300
C5—H5	0.9300	C18—C19	1.3950 (16)
C6—H6	0.9300	C18—C23	1.4000 (15)
C7—N2	1.3084 (15)	C18—N3	1.4102 (14)
C7—C9	1.4403 (15)	C19—C20	1.3892 (16)
C7—C8	1.4928 (16)	C19—H19	0.9300
C8—H8A	0.9600	C20—C21	1.3900 (18)
C8—H8B	0.9600	C20—H20	0.9300
C8—H8C	0.9600	C21—C22	1.3863 (17)
C9—C11	1.3887 (15)	C21—H21	0.9300
C9—C10	1.4526 (16)	C22—C23	1.3870 (15)
C10—O1	1.2464 (14)	C22—H22	0.9300
C10—N1	1.3784 (14)	C23—Cl1	1.7384 (11)
C11—N3	1.3445 (14)	N1—N2	1.4012 (13)
C11—C12	1.4855 (15)	N3—H3A	0.877 (19)
C12—C17	1.3925 (16)		
			
C2—C1—C6	120.02 (11)	C14—C13—H13	120.1
C2—C1—N1	119.96 (11)	C12—C13—H13	120.1
C6—C1—N1	120.02 (10)	C15—C14—C13	120.10 (13)
C1—C2—C3	119.47 (12)	C15—C14—H14	120.0
C1—C2—H2	120.3	C13—C14—H14	120.0
C3—C2—H2	120.3	C14—C15—C16	120.34 (12)
C4—C3—C2	120.65 (12)	C14—C15—H15	119.8
C4—C3—H3	119.7	C16—C15—H15	119.8
C2—C3—H3	119.7	C15—C16—C17	120.11 (13)
C5—C4—C3	119.39 (12)	C15—C16—H16	119.9
C5—C4—H4	120.3	C17—C16—H16	119.9
C3—C4—H4	120.3	C16—C17—C12	119.73 (12)
C4—C5—C6	120.78 (12)	C16—C17—H17	120.1
C4—C5—H5	119.6	C12—C17—H17	120.1
C6—C5—H5	119.6	C19—C18—C23	118.37 (10)
C5—C6—C1	119.50 (12)	C19—C18—N3	122.96 (10)
C5—C6—H6	120.2	C23—C18—N3	118.51 (10)
C1—C6—H6	120.2	C20—C19—C18	120.23 (11)
N2—C7—C9	111.56 (10)	C20—C19—H19	119.9
N2—C7—C8	118.52 (10)	C18—C19—H19	119.9
C9—C7—C8	129.90 (10)	C19—C20—C21	120.61 (11)
C7—C8—H8A	109.5	C19—C20—H20	119.7
C7—C8—H8B	109.5	C21—C20—H20	119.7
H8A—C8—H8B	109.5	C22—C21—C20	119.87 (11)
C7—C8—H8C	109.5	C22—C21—H21	120.1
H8A—C8—H8C	109.5	C20—C21—H21	120.1
H8B—C8—H8C	109.5	C21—C22—C23	119.42 (11)
C11—C9—C7	132.45 (11)	C21—C22—H22	120.3
C11—C9—C10	122.12 (10)	C23—C22—H22	120.3
C7—C9—C10	105.11 (10)	C22—C23—C18	121.49 (10)
O1—C10—N1	126.82 (11)	C22—C23—Cl1	119.26 (9)
O1—C10—C9	128.70 (11)	C18—C23—Cl1	119.25 (9)
N1—C10—C9	104.48 (9)	C10—N1—N2	112.22 (9)
N3—C11—C9	117.88 (10)	C10—N1—C1	128.54 (10)
N3—C11—C12	120.12 (10)	N2—N1—C1	119.16 (9)
C9—C11—C12	121.87 (10)	C7—N2—N1	106.62 (9)
C17—C12—C13	119.80 (11)	C11—N3—C18	129.41 (10)
C17—C12—C11	119.38 (10)	C11—N3—H3A	113.1 (12)
C13—C12—C11	120.77 (10)	C18—N3—H3A	117.4 (12)
C14—C13—C12	119.87 (13)		
			
C6—C1—C2—C3	3.56 (19)	C13—C12—C17—C16	−2.44 (17)
N1—C1—C2—C3	−175.90 (12)	C11—C12—C17—C16	174.82 (11)
C1—C2—C3—C4	0.2 (2)	C23—C18—C19—C20	−0.05 (17)
C2—C3—C4—C5	−3.0 (2)	N3—C18—C19—C20	175.29 (11)
C3—C4—C5—C6	2.1 (2)	C18—C19—C20—C21	−0.23 (18)
C4—C5—C6—C1	1.6 (2)	C19—C20—C21—C22	0.31 (18)
C2—C1—C6—C5	−4.49 (19)	C20—C21—C22—C23	−0.10 (17)
N1—C1—C6—C5	174.98 (12)	C21—C22—C23—C18	−0.18 (17)
N2—C7—C9—C11	174.02 (13)	C21—C22—C23—Cl1	−179.40 (9)
C8—C7—C9—C11	−4.3 (2)	C19—C18—C23—C22	0.26 (17)
N2—C7—C9—C10	0.57 (14)	N3—C18—C23—C22	−175.30 (10)
C8—C7—C9—C10	−177.72 (13)	C19—C18—C23—Cl1	179.47 (8)
C11—C9—C10—O1	5.5 (2)	N3—C18—C23—Cl1	3.92 (14)
C7—C9—C10—O1	179.79 (13)	O1—C10—N1—N2	179.70 (12)
C11—C9—C10—N1	−174.35 (11)	C9—C10—N1—N2	−0.45 (13)
C7—C9—C10—N1	−0.05 (13)	O1—C10—N1—C1	3.2 (2)
C7—C9—C11—N3	−170.78 (12)	C9—C10—N1—C1	−176.98 (11)
C10—C9—C11—N3	1.74 (17)	C2—C1—N1—C10	161.96 (12)
C7—C9—C11—C12	5.1 (2)	C6—C1—N1—C10	−17.50 (19)
C10—C9—C11—C12	177.64 (11)	C2—C1—N1—N2	−14.36 (17)
N3—C11—C12—C17	63.52 (15)	C6—C1—N1—N2	166.17 (11)
C9—C11—C12—C17	−112.29 (13)	C9—C7—N2—N1	−0.83 (14)
N3—C11—C12—C13	−119.24 (13)	C8—C7—N2—N1	177.68 (11)
C9—C11—C12—C13	64.95 (15)	C10—N1—N2—C7	0.81 (14)
C17—C12—C13—C14	0.82 (18)	C1—N1—N2—C7	177.71 (11)
C11—C12—C13—C14	−176.41 (11)	C9—C11—N3—C18	−175.06 (11)
C12—C13—C14—C15	1.23 (19)	C12—C11—N3—C18	8.97 (18)
C13—C14—C15—C16	−1.7 (2)	C19—C18—N3—C11	37.69 (18)
C14—C15—C16—C17	0.0 (2)	C23—C18—N3—C11	−146.98 (12)
C15—C16—C17—C12	2.03 (18)		

### Hydrogen-bond geometry (Å, º)

**Table:**

*D*—H···*A*	*D*—H	H···*A*	*D*···*A*	*D*—H···*A*
N3—H3*A*···O1	0.879 (18)	1.915 (18)	2.6800 (14)	144.5 (16)
C6—H6···O1	0.93	2.31	2.9027 (16)	121
C16—H16···O1^i^	0.93	2.56	3.4797 (17)	170

Symmetry code: (i) −*x*+3/2, *y*+1/2, −*z*+1/2.
